# Practical Notes and Formulæ

**Published:** 1888-06

**Authors:** 


### PRACTICAL NOTES AND FORMULÆ.

**Cascara Sagrada.**—Dr. L. L. Clement writes: If I had to use one remedy to the exclusion of all others, I should use cascara sagrada. When there is accumulation of gas and the food is causing distress, lying in the stomach rotting, if you will give a few drops of the fl. ext., it causes eructation of gas, starts the stomach to action, and the patient experiences relief in most cases, and will not do without the remedy after giving it a fair trial. A sufficient quantity of the fl. ext. causes a natural evacuation of the bowels; and in small doses it seems to have a soothing effect on the mucous membrane of the stomach.

**Dysentery.**—

℞. Acidi muratici deluti....................... ʒ j,

Morphiæ sulph......................... gr. ij,

Aqua distillatæ............................ ℥ iv.

M. S.—Take one teaspoonful every four to six hours in wineglassful of water, previously clearing the bowels with a dose of epsom salts.

**Dysentery.**—

℞. Tinc. belladonna............................. ʒ j,

Water...................................... ℥ viij.

M. Teaspoonful every hour until pupils dilate, then lengthen the interval. This prescription to be preceded by calomel, gr. v, worked off with a dose of siedlitz powders.

**Pruritus Ani.**—Dr. W. P. Agnew asks for some "pointers on pruritus ani." The following treatment is efficient in many cases:

℞. Acidi carbolici.......................... ʒ ij,

Glycerini.. ............................ ℥ ss,

Aquæ q. s. ad.......................... ℥ viij.

M. Sig. Apply to the parts with a cloth when there is much itching.

℞. Hydrarg. ammoniati....................... ʒ j,

Ung. zinci oxidi.......................... ℥ j.

M. S.—Apply morning and night.—*Dr. A. G. Craig, in Med. World.*

**Pile Ointment.**—For many years I have used the following with unvarying satisfaction. I have now no distinct recollection to whom credit for it should be given.

℞. Ext. opii aquosi ........................ gr. x,
Acidi tannici ........................ ʒ j,
Cerat. plumbi subacetat ........................ ℥,

M. et. ft. ungt. S.—Apply morning and night.—*Dr. Ben. Edson, in Medical World.*

**Geranium Maculatum in Chronic Bronchitis.**—*The Journal de Medecine*, of March 11, 1888, gives the following as a useful prescription in chronic bronchitis and bronchorrhœa:

℞. Tinct. nucis vom ........................ ʒ j,

Tinct. sanguinariæ ........................ ʒ j,

Ext. fluid. geranii ........................ ʒ jss,

Syrup. simp ........................ ℥ iv.

Of this a drachm should be taken every four hours.—*Medical News, April 14, 1888.*

**Convenient Combinations for Administering Hydrochloric Acid in Dyspepsia.**—The *Revue Generale de Clinique et de Therapeutique*, of February 9, 1888, contains a number of formulæ for the administration of hydrochloric acid from which we take the following:

Hydrochloric acid lemonade:

℞. Aquæ ........................ O i¾,

Syrup. simpl ........................ ʒ xxv,

Acid. hydrochlor ........................ ♏xxx to lx,

Spirit, limonis ........................ ♏ xv.

A half glass or glass after meals.

A digestive and laxative potion may be ordered as follows:

℞. Acid. hydrochloric ........................ ♏ xv,

Syrup simpl ........................ ʒ viiiss,

Vini rhei ........................ ʒ xxv.

Of which the dose may be varied as indications demand.

West prescribed for children the following mixture, in doses of one to two teaspoonfuls:

℞. Acid. hydrochloric, dil ........................ ♏ xv,

Syrup. aurant. cortic ........................ ♏ lxxxv.

Tinct. aurant. amari ........................ ♏ xlv,

Infusion. cascarillæ ........................ ʒ x.

Hydrochloric acid is given to increase the acidity of the gastric juice; to dissolve phosphates and alkalies; to contribute to the formation of chloride of sodium in the organism. In general terms, it is given before meals to assist in overcoming gastric atony; after meals to remedy hyper-alkalescence.—*Medical News*.

